# Effects of *Atractylodes lancea* extracts on intestinal flora and serum metabolites in mice with intestinal dysbacteriosis

**DOI:** 10.1186/s12953-023-00204-x

**Published:** 2023-04-15

**Authors:** BaiNian Zhang, Lan Bu, Hui Tian, ZhangQiang You, MingHai Zhao, Jie Tian, YuanYuan Zhang, Qian Wang, ChengJia Tan, Yu Cao, DaRen Feng, ZhenPeng Xi

**Affiliations:** 1 Key Laboratory of Quality Control of Traditional Chinese Medicine in Northwest Sichuan, Mianyang Normal University, Mianyang, 621000 China; 2 Mianyang Institute for Food and Drug Control, Mianyang, 621000 China; 3 Beichuan Shennong Agriculture Technology Development Co.,Ltd, Mianyang, 621000 China

**Keywords:** *Atractylodes lancea* extract, Gut microbiota, Metabolomics

## Abstract

**Objective:**

This study aims to explore the effect of an extract of *Atractylodes lancea* (*A. lancea*) on antibiotics-induced intestinal tract disorder and the probable therapeutic mechanisms employed by this extract to ameliorate these disorders.

**Methods:**

Three days after acclimatization, nine male and nine female specific-pathogen-free (SPF) mice were randomly assigned into three groups: Group C (normal saline), Group M (antibiotic: cefradine + gentamicin), and Group T (antibiotic + *A. lancea* extract). Each mouse in Groups M and T received intragastric (i.g.) gavage antibiotics containing cefradine and gentamicin sulfate (0.02 ml/g^−1^/D^−1^) for 7 days. *A. lancea* extract (0.02 ml/g^−1^/D^−1^) was administered by i.g. gavage to Group T mice for 7 days following the cessation of antibiotic therapy. Group M received an equivalent volume of normal saline for 7 days, while Group C received an equivalent volume of normal saline for 14 days. Afterwards, we collected mouse feces to assess changes in intestinal microbiota by 16S ribosomal ribonucleic acid (rRNA) sequencing and metabolomics. In addition, serum samples were gathered and analyzed using liquid chromatography–mass spectrometry (LS-MS). Finally, we performed a correlation analysis between intestinal microbiota and metabolites.

**Results:**

After treatment with antibiotic, the richness and diversity of the flora, numbers of wall-breaking bacteria and Bacteroidetes, and the numbers of beneficial bacteria decreased, while the numbers of harmful bacteria increased. After i.g. administration of *A. lancea* extract, the imbalance of microbial flora began to recover. Antibiotics primarily influence the metabolism of lipids, steroids, peptides, organic acids, and carbohydrates, with lipid compounds ranking first. Arachidonic acid (AA), arginine, and proline have relatively strong effects on the metabolisms of antibiotic-stressed mice. Our findings revealed that *A. lancea* extract might restore the metabolism of AA and L-methionine. The content of differential metabolites detected in the serum of Group T mice was comparable to that in the serum of Group C mice, but significantly different from that of Group M mice. Compared to putative biomarkers in the Kyoto Encyclopedia of Genes and Genomes (KEGG) database, it was found that altered metabolites, such as amino acids, glycerol, and phospholipids, were primarily associated with the metabolism.

**Conclusions:**

The effective mechanisms of *A. lancea* extract in regulating the disorder of intestinal flora in mice are related to the mechanisms of *A. lancea*. It could relate to lipid metabolism, bile acid metabolism, and amino acid metabolism. These results will provide a basis for further explaining the mechanism by which *A. lancea* regulats intestinal flora.

## Introduction

The gut microbiome is an essential microecosystem for the host’s health and metabolic equilibrium [1]. It is crucial to maintain the microenvironmental balance of the gut microbiota [2]. When the balance is disrupted by external factors such as food poisoning [3, 4] and antibiotic abuse [5–8], the host’s intestinal flora is disturbed and metabolic level and health are impacted, resulting in decreased immune function, impaired internal organs and bone marrow hematopoietic function, leading to anemia and other diseases. In recent years, numerous studies have demonstrated that traditional Chinese medicine (TCM) can stimulate the proliferation of probiotics, limit the growth of harmful pathogenic, and preserve the gut microbiota’s balance [9].

Atractylodis Rhizoma is a perennial herb of the family Compositae [10]. There are around seven species in the genus *Atractylodes*, which are primarily found in eastern Asia. There are five species in China, such as *A. lancea*, *A. macrocephala*, *A. carlinoides*, *A. koreana*, and *A. japonica*. The dried rhizomes of *Atractylodes lancea* or *Atractylodes chinensis* are referred to as Atractylodis Rhizoma in the 2020 edition of the Chinese Pharmacopoeia. This substance is widely available throughout China. Several Atractylodes species are utilized instead of *A. carlinoides* for clinical therapeutic uses. This article uses *A. lancea* [11] exerting various pharmacological effects, which are applied widely in clinical practice, such as treating gastric ulcers [12–15], tumors, and inflammation [16], protecting the liver [17], regulating gastrointestinal (GI) activities [18], and inhibiting gastric acid secretion. It is particularly effective in the treatment of GI diseases. Researches on its pharmacological action in treating various disorders have been conducted at home and abroad [19]. However, there are very few investigations on the mechanism by which *A. lancea* heals gastrointestinal diseases.

Combining 16S ribosomal ribonucleic acid (rRNA) microbial-diversity sequencing [20, 21] and broadly targeted metabolomics [22–24] is a typical method to investigate suspected metabolic disorders and drug mechanisms nowadays. The sequencing of Microbial diversity based on 16S rRNA is frequently used to detect changes in microbial-community diversity and species richness and can categorize bacteria rapidly and precisely. As a new “omics” field, metabolomics [25] possesses the characteristics of integrity, dynamicicity, and non-targeting, and is a potent research tool for chronic metabolic diseases. It can reflect the metabolic pathways and networks of biological samples and identify metabolic anomalies at the molecular level that caused by disease. Typically, stool and serum are utilized as samples to examine changes in the variety of gut microbiota and metabolic differences before and after an experiment [26].

Consequently, the purpose of this study is to detect changes in intestinal flora using 16S rRNA sequencing when drugs induce intestinal flora disorder in mice. Our objectives were to conduct serum metabolomics investigations centered on metabolites, evaluate the effect of *A. lancea* extract on intestinal-microbiota problem, and analyze its therapeutic mechanism in mice based on alterations in endogenous chemicals [27].

## Experimental materials

### Experimental animals

We obtained a total of 18 healthy specific-pathogen-free (SPF) mice from Chengdu Dashuo Animal Co. Ltd. (Chengdu, China). The weight varied between 17.0 and 22.0 g.

### Experimental drugs and reagents

*A. lancea* (with 0.30% atractydin) was bought from Chengdu Durst Biotechnology Co., Ltd. (Chengdu, China). Cefradine capsules were bought from Hunan Kelun Pharmaceutical Co., Ltd. (Chengguan Town, China; State Food and Drug Administration (SFDA) Approval No. H43022215; specification: 0.5 g). Gentamycin sulfate was purchased from Huazhong Pharma Co., Ltd. (Shenzhen, China, SFDA Approval No. H42021503; specification: 2 ml: 0.08 g (80,000 units)). E.Z.N.A. Soil Kit (Omega Bio-tek, Inc., Norcross, GA, USA).

### Experimental equipment

Thermo Cyclist PCR Analysis System (GeneAm9700) was purchased from Affinity Biosciences, Inc. (Cincinnati, OH, USA). The AxyPrep Deoxyribonucleic Acid (DNA) Gel Extraction Kit was purchased from Axygen Biosciences (Union City, CA, USA). The QuantiFluor-ST Double-stranded DNA (dsDNA) System was purchased from Promega Corp. (Fitchburg, WI, USA). An ultra-low–temperature freezer was purchased from Thermo Fisher Scientific (No. FUMA-86C). Liquid chromatography–mass spectrometry (LC–MS) methanol was purchased from Thermo Fisher Scientific (Moscow, Russia; Cat. No. A456-4). LC–MS acetonitrile was purchased from Thermo Fisher Scientific (Moscow, Russia; Cat. No. A955-4). LC–MS formic acid was purchased from Thermo Fisher Scientific (Moscow, Russia; Cat. No. A117-50). LC–MS water was purchased from Thermo Fisher Scientific (Moscow, Russia; Cat. No. W6-4). High-performance liquid chromatography (HPLC) 2-propanol was purchased from Thermo Fisher Scientific (Moscow, Russia; Cat. No. A451-4), NanoDrop2000.

## Experimental method

### Grouping and numbering of mice, and preparation of intestinal-dysbiosis mouse model

**Fig. 1:**
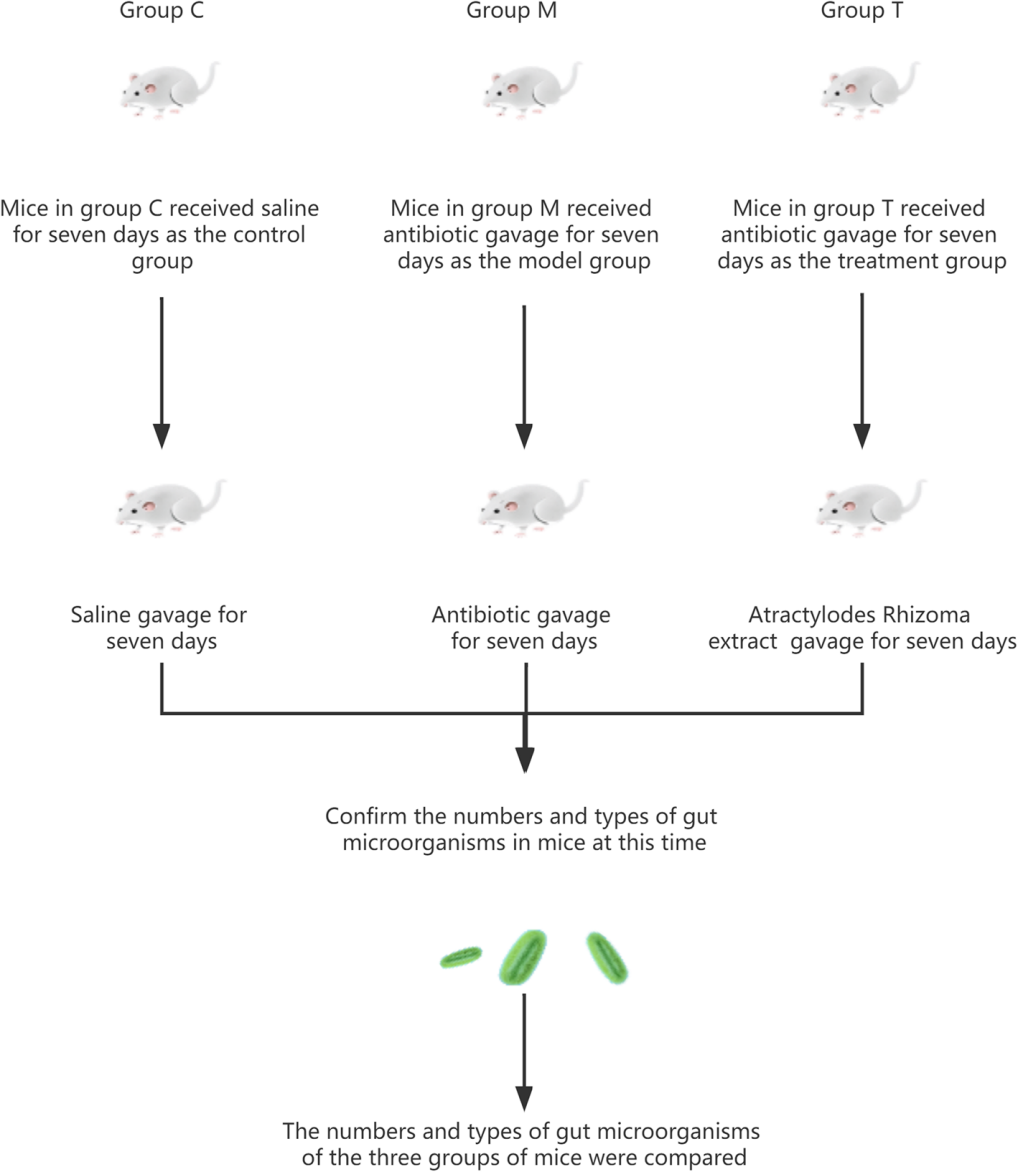
The experimental design and process

The mice were domesticated for 3 days before being randomly separated into three groups: C (normal saline), M (antibiotic: cefradine + gentamicin), and T (cefradine + gentamicin + *A. lancea*). They were designated by the designations C1–C6, M1–M6, and T1–T6. For modeling, each mouse in Groups M and T was administered 0.02 ml/g1/D1 of cefradine + gentamicin sulfate by intragastric (i.g.) gavage for seven days. Group C received an equivalent volume of normal saline by i.g. gavage for 7 days. At the end of the experiment, we collected mouse feces and preserved by freezing them in a cryogenic refrigerator. Figure 1 shows the experimental process.

### Drug treatment

Group T mice were given *A. lancea* extract (0.02 ml/g^−1^/D^−1^) by i.g. gavage for 7 days, while Groups M and C were given normal saline.

### Sample collection

#### Feces

Mouse feces from the last 3 days of the experiment were collected and stored in a sterile Eppendorf tube at − 80 °C.

#### Serum

On the final day of the experiment, we used the lavage needle to collect blood from the mice's abdominal cavities. The blood was kept on ice for 30 min and then centrifuged at 4 °C and 3000 g for 15 min. Afterwards, we extracted the supernatant (serum) and stored it in a freezer at − 80 °C.

### Treatment of fecal samples (16S rRNA diversity sequencing)

#### Deoxyribonucleic acid extraction and polymerase chain reaction amplification

DNA was extracted from the whole flora of the mouse feces following the instructions of an E.Z.N.A. Soil Kit. The concentration and purity of extracted DNA were determined using a NanoDrop 2000, and the DNA's quality was determined using 1% agarose gel electrophoresis. We amplified the V3–V4 variable region by polymerase chain reaction (PCR) using 806R and 338F primers.

#### Diversity sequencing

PCR products were extracted from 2% agarose gel, purified, analyzed, and quantified using the QuantiFluor-ST. The purified amplified sequences were made into a 2 × 300 paired-end (PE) library using an Illumina MiSeq platform and sequenced on a MiSeq PE300 platform.

### Treatment of serum samples

#### Pretreatment

We moved 100 µL of plasma sample with precision and added 20 µL of internal standards (0.3 mg/ml L-2-chlorophenylalanine + acetonitrile). Subsequently, we added 400 µL extract (methanol:acetonitrile = 1:1) to the sample, mixed it in a vortex mixer for 30 s, and performed low-temperature ultrasonic extraction for 30 min at 5 °C and 40 kHz. Then, the sample was frozen at − 20 °C for 30 min and centrifuged for 15 min (13,000 g, 4 °C), then the supernatant liquid was removed and blown dry with nitrogen gas. Finally, 100 µL complex solution (acetonitrile:water = 1:1) underwent low-temperature ultrasonic extraction for 5 min (5 °C, 40 kHz) and then centrifugation for 5 min (13,000 g, 4 °C), after which the supernatant was transferred to the sample bottle for machine detection.

#### LC–MS detection

The instrument used for LC–MS analysis was an AB SCIEX Ultra-HPLC (UHPLC) TripleTOF System.

*Chromatographic conditions:* The chromatography column was a BEH C_18_; mobile phase A was water (containing 0.1% formic acid), while mobile phase B was acetonitrile: isopropanol (1:1; containing 0.1% formic acid). The flow rate was 0.40 ml/min, coupled with a 10 μL injection volume and a 40 °C column temperature.

*MS conditions:* We used positive-ion and negative-ion scanning mode, as well as ion spray voltage for sample quality spectrum signal acquisition.

See Table 1 for specific parameters.

**Table 1:** Source gas parameters and impact energy of mass spectrometry

Mass range	m/z	Parameter
Spray gas	Ion source gas 1 (psi)	50
Auxiliary heating	Ion source gas 2 (psi)	50
Gas curtain	Curtain gas (psi)	30
Heating temperature source	Source temperature (°C)	500
Ionization voltage ( +)	Ion spray voltage floating (electrospray ionization [ESI]^+^)(V)	+ 5000
Ionization voltage ( −)	Ion spray voltage floating (ESI^−^)(V)	− 4000
Voltage clustering	Declustering potential (V)	80
Impact energy	MS–MS collision energy (V)	20–60 (rolling)

### Data analysis

#### Intestinal-flora analysis

In the experiment, the original sequence from the MiSeq PE300 was checked for quality (QC) with Trimmomatic. Fast Length Adjustment of SHort Reads (FLASH) (Magoč and Salzberg, 2011) was used to optimize and screen bacterial 16S rRNA data. The experiment began by setting a 50-bp window. When average quality was < 20, all sequence fragments at the back end of the base were removed from the front of the window, and then sequence fragments < 50 bp long after QC were removed. The experiment spliced two sequences based on overlap (maximum error matching rate = 0.2; length > 10 bp). According to the barcode and primers at the beginning and end of the sequence, we split the sequence for each sample. Finally, UPARSE (Edgar, 2013) was used to perform an operational taxonomic-unit (OTU) clustering analysis on the sequence based on 97% similarity.

#### Serum metabolite analysis

The data were initially preprocessed. We imported the raw data into the metabolomics processing software Progenesis QI (Waters) for baseline filtering, peak recognition, integration, retention time correction, and peak alignment. Ultimately, a data matrix containing retention time, mass charge ratio, and peak strength was acquired. Next, the accompanying operations were performed: (1) retaining > 80% of nonzero variables in the sample; (2) supplementing the minimum value missing from the original matrix; (3) normalizing the total peak; and (4) obtaining the data matrix via log transformation for further analysis. Then, we performed unsupervised principal component analysis (PCA) and supervised orthogonal partial least squares discriminant analysis (OPLS-DA). Student’s *t* test and variable importance in projection (VIP) were coupled to identify differential metabolites, followed by differential-metabolite cluster analysis and Kyoto Encyclopedia of Genes and Genomes (KEGG) functional pathway, pathway enrichment, and topological analyses. The biological data of several metabolites were extracted.

## Experimental results

### Intestinal-flora analysis

**Fig. 2:**
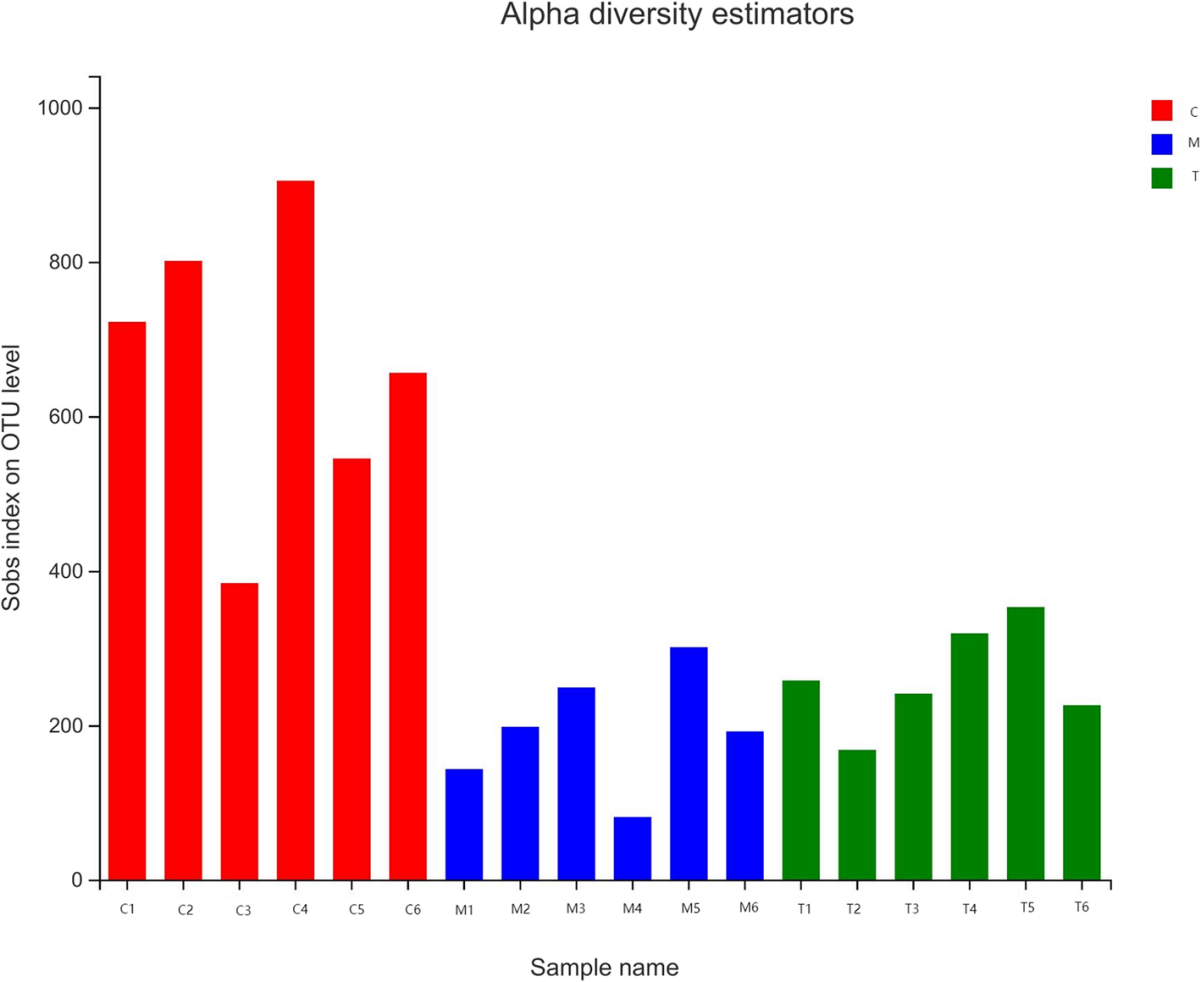
Diversity index. The abscissa is the sample, and the ordinate is the number of species observed at the genus level

**Fig. 3:**
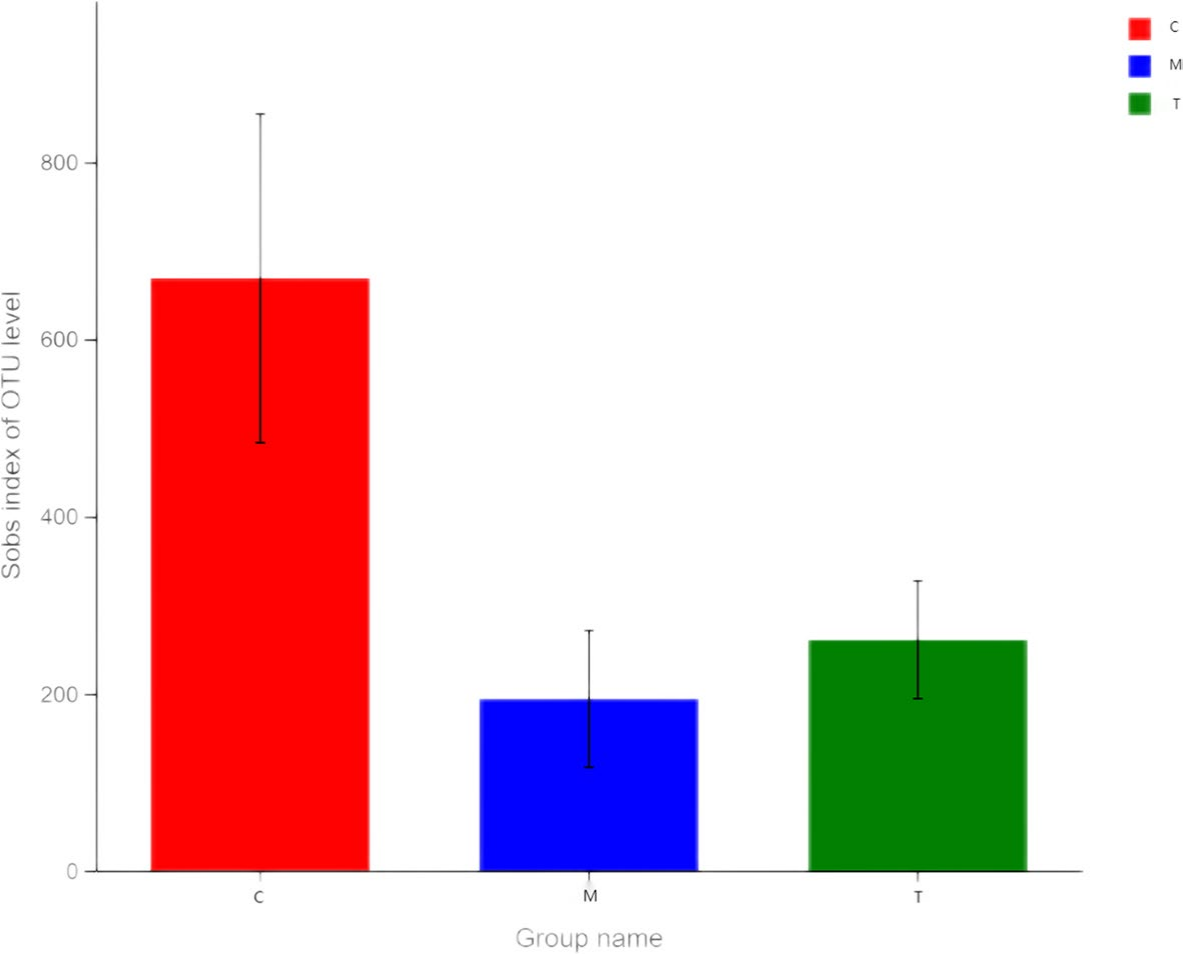
Student’s *t* test histogram of intergroup differences. The abscissa is the group name, and the ordinate is the mean value of Shannon index at the OUT classification level. (*P < 0.05, **P < 0.01, ***P < 0.001)

The observed species (Sobs) index can be used to judge the richness of microbial colonies, while the Shannon index can be used to judge the diversity of such colonies. The number of microorganisms in the large intestines of mice that were gagged with normal saline is what "Group C" means. After the gavage of antibiotics, the diversity of organisms was greatest in Group M. As can be seen, compared with Group C, the species diversity and richness of the intestinal microbial community in Group M were lower. The T group reveals that specific bacteria exhibited a pattern of recovery and expansion (Figs. 2 and 3). After administration of *A. lancea* extract, the diversity and abundance of microorganisms in the large intestines of mice tended to recover and grow.

**Fig. 4:**
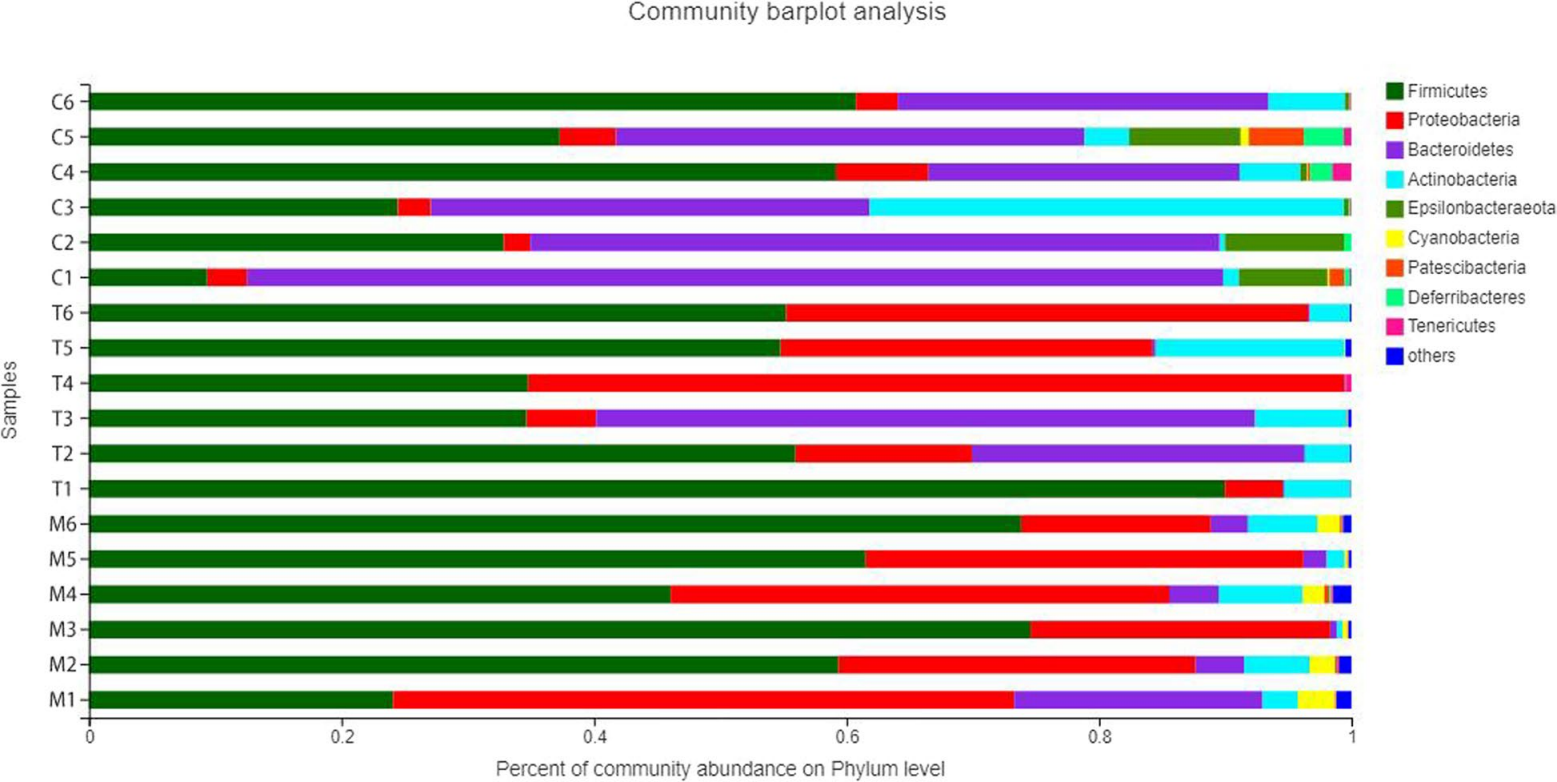
Histogram of sample community composition. The abscissa is the proportion of species, and the ordinate is the sample number

The bar diagram in Fig. 4 indicates the compositions of numerous phyla at various taxonomic levels, as well as the corresponding dominant phyla and their proportions. At the phylum level, the biggest proportion of bacteria among the three groups was Firmicutes. The proportion of Bacteroides reduced considerably after antibiotic gavage, and Proteobacteria replaced it as the dominating phylum. Following treatment with *A. lancea* extract, Bacteroides and Actinomycetes proportions increased. When antibiotics were administered to mice, the phyla of bacterial in their intestines changed. The percentage of probiotics decreased. The equilibrium of flora was upset, and the usual growth of helpful beneficial bacteria was impeded. However, after treatment with *A. lancea* extract, the number of beneficial bacteria began to recover, and the proportion of harmful bacteria tended to return to its level prior to antibiotic administration, indicating that *A. lancea* extract had a significant effect on restoring the balance of intestinal flora in mice.

**Fig. 5:**
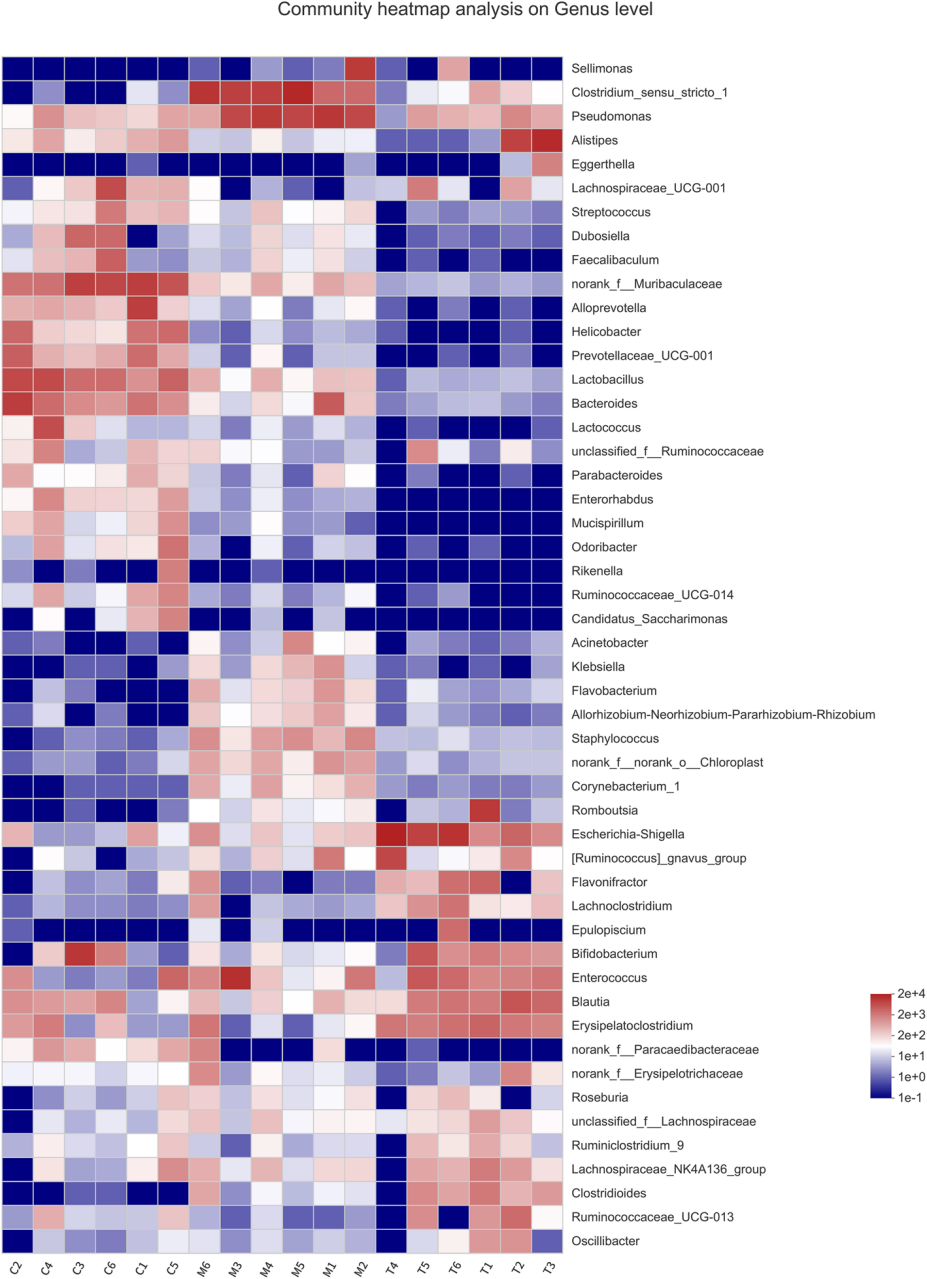
Heatmap of sample community. The abscissa is the sample number, the ordinate is the species name, and the legend is the species abundance value

For heatmap analysis, the 50 genera with the highest abundance were screened. The genera *Polyformis*, *Prevosiella*, and *Muribaculaceae* were more abundant in the normal group, as depicted by the community heatmap at the genus level (Fig. 5). Antibiotics decreased the number of bacteria in the normal group, but *Pseudomonas aeruginosa*, *Enterococcus*, and *Clostridium* increased in number. The leading colonies were *Escherichia Shigella* and *Otherbacter*, and several of the more abundant communities in the normal group were recovering. However, the abundance of some medications was lower than that of antibiotics (Group A), which may have been due to the brief time of oral *A. lancea* extract administration and the ease with which other microorganisms were recovered from the environment formed by *A. lancea*. In contrast, several bacteria were more sensitive to the environment generated by antibiotics or *A. lancea*..

**Fig. 6:**
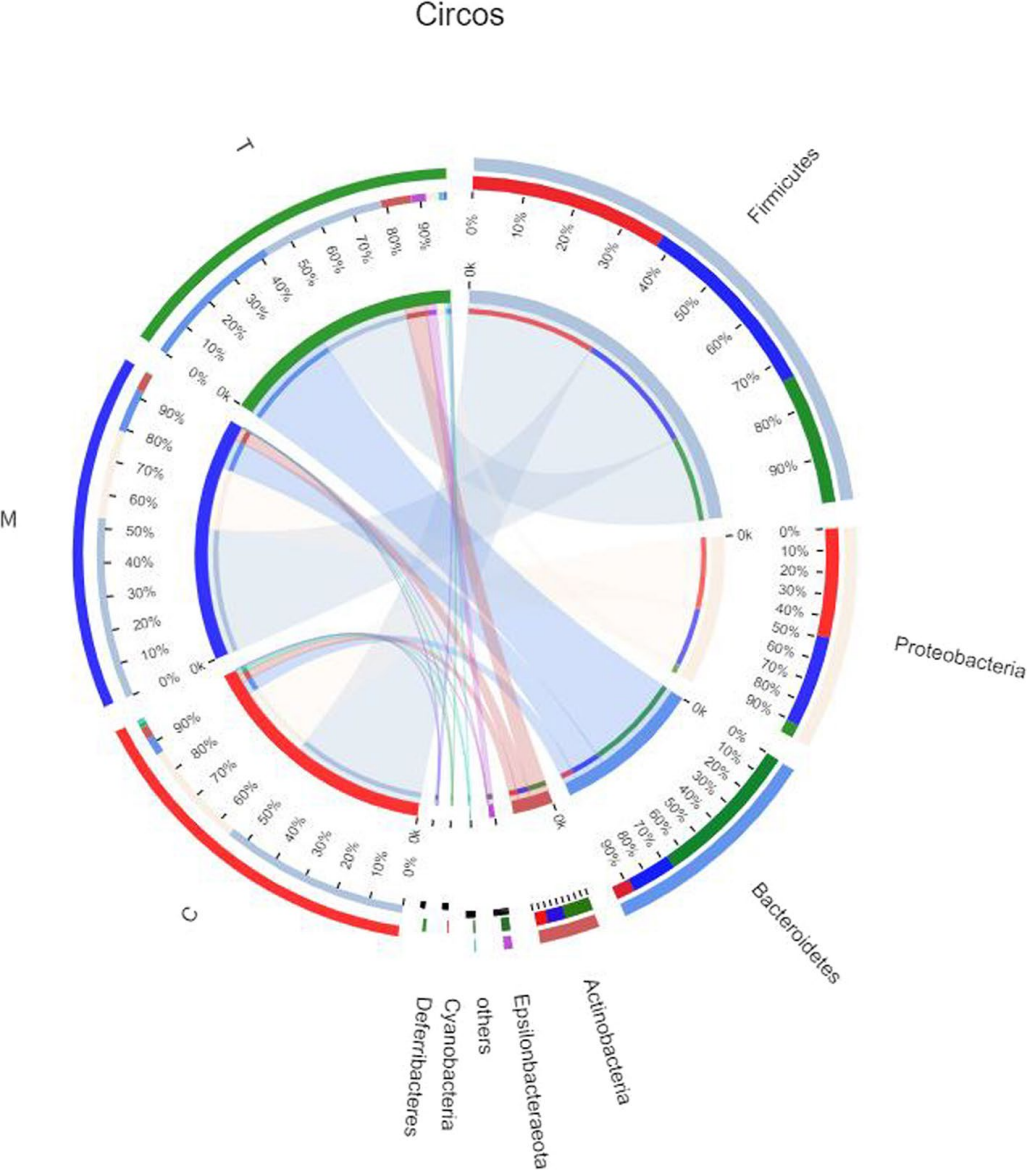
Circos diagram. In the Circos sample and species diagram, the large semicircle (right half circle) represents the distribution proportion of a species in different samples under a certain classification level (outer ribbon: species, inner ribbon color: different groups), and the length represents the specific distribution proportion; The small semicircle (left half circle) represents the composition of different species in a sample (outer band: grouping, inner band: species), and the relative abundance specific to length

We utilized a Circos diagram (Fig. 6) to examine the composite proportions of dominant phyla in various samples. Under normal conditions, Firmicutes and Bacteroidetes are the dominant groups. In mice treated with antibiotics, Proteobacteria grew from 3.8% to 32%, whereas Bacteroidetes declined from 43% to 5.5%, demonstrating that antibiotics can alter the composition of the intestinal flora and hence produce an intestinal-flora problem. After i.g. treatment with *A. lancea* extract, Mycorrhizae and Bacteroidetes were once again in the dominating floras. The drop in Proteobacteria and significant rise in Bacteroidetes (from 5.5% to 13%) demonstrated that *A. lancea* extract might enhance the number of beneficial bacteria in the digestive tract and restore the balance of intestinal flora.

**Fig. 7:**
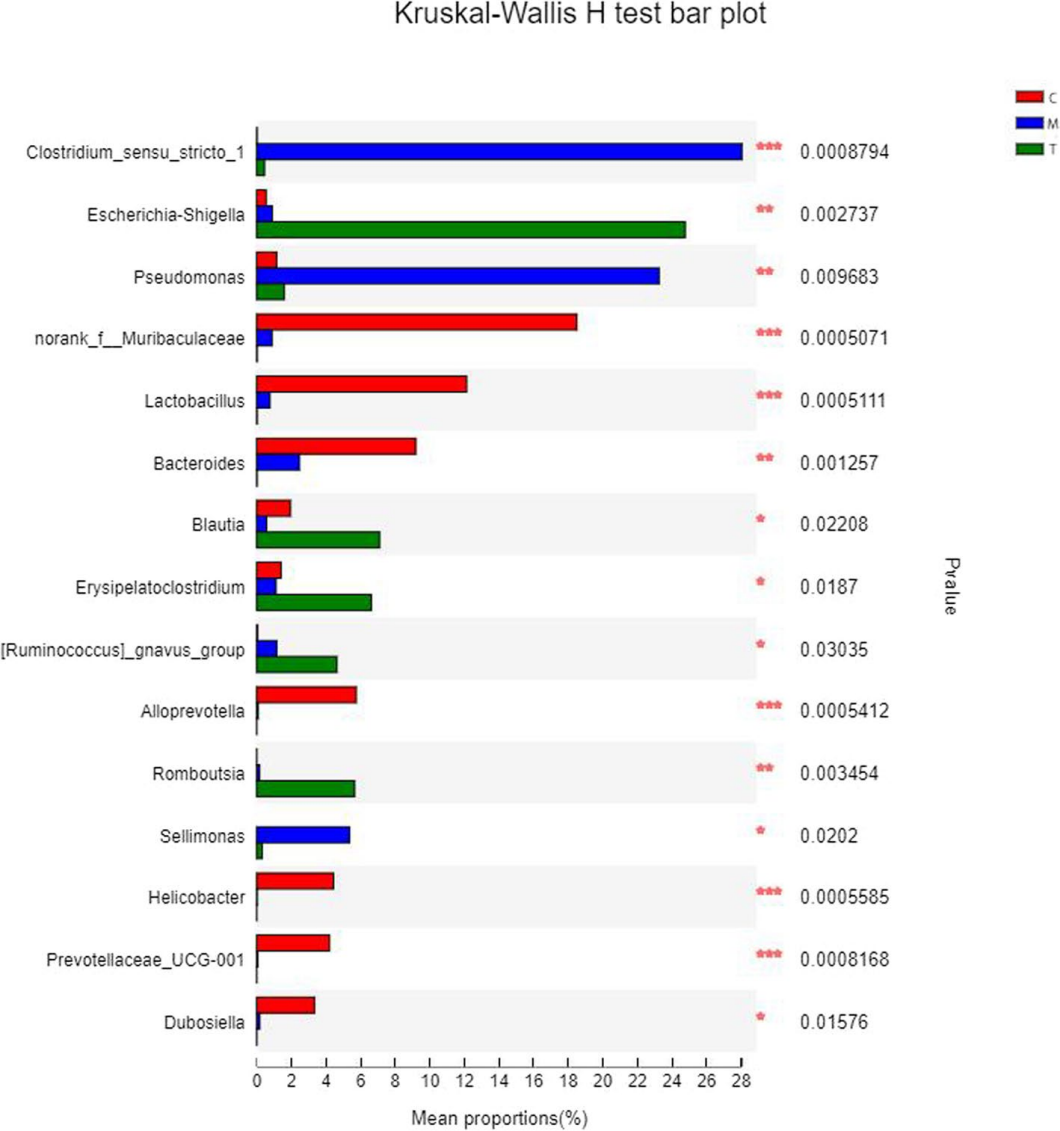
Multi-group comparison of significant differences between groups. The left column shows the species name under the genus classification, and the corresponding column shows the average relative abundance of the species in each sample group. Different groups have different colors. The right-hand side is the *p*-value. (*P < 0.05, **P < 0.01, ***P < 0.001)

We used the Kruskal–Wallis *H* test (Fig. 7) to assess statistically differences between groups and to determine differences in species richness within each group. As shown in Fig. 7, the three groupings included *Muribaculaceae*, *Clostridium*, *Lactobacillus*, *Prevotella* (*P* ≤ 0.001), *Eschia*, *P. aeruginosa*, *multiform rod-shaped bacteria*, and *Romboutsia* (0.001 < *P* ≤ 0.01). There were significant differences between the eight different genera. In addition, the color columns of the three groups indicated that the species richness of Group C, which was initially low, increased after the administration of antibiotics. Examples include *P. aeruginosa* and *Clostridium*. These findings demonstrated that antibiotics can alter the equilibrium of intestinal flora. Following treatment with *A. lancea* extract, the relative abundance of these two species fell on average. These results demonstrated that *A. lancea* extract may restore the average relative abundance of intestinal microflora and treat intestinal dysbiosis in mice.

**Fig. 8:**
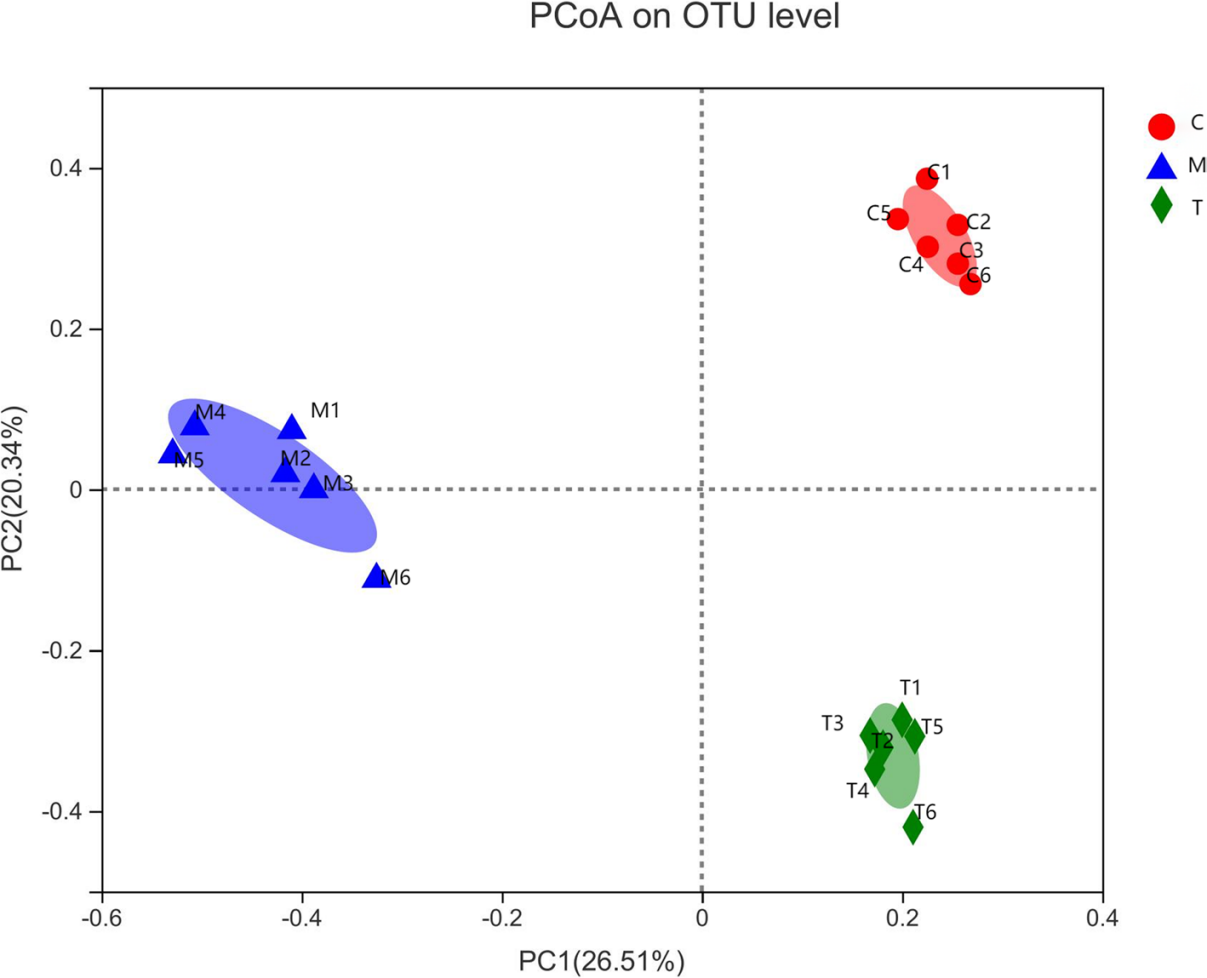
Principal component analysis. The closer the two sample points are, the more similar the species composition of the two samples is

In addition, PCA based on OTUs indicated that these populations had distinct microbiome characteristics. As represented in Fig. 8, the results from the three groups showed a distinct difference phenomenon, confirming the accuracy of our experimental methodology. At the same time, mouse A6 in Group A showed obvious separation from other members of the same group, which have been attributable to unique causes.

**Fig. 9:**
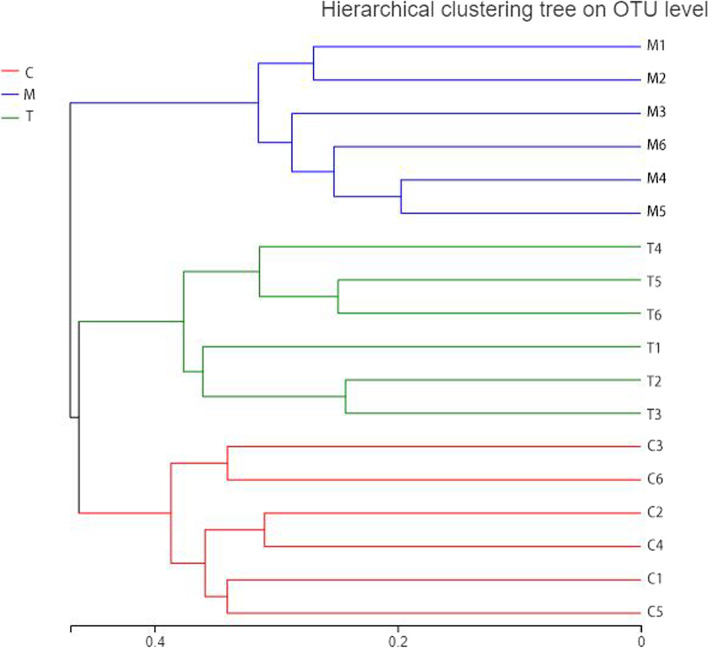
Significance of differences between groups. Clustering tree graph for multi-group comparisons between samples

Significant separation was also observed in the hierarchical-clustering results (Fig. 9), which was consistent with PCA results. Meanwhile, the three classifications shown in Fig. 9 are unique, indicating that the test technique and grouping strategy we picked were appropriate. The performance of test objects in subgroups can be seen from the data set of Group C and Group A_A (Group T), but mouse A6 in Group A is clustered distant from the other members of the same group in the PCA graph due to its individual differences.

The abovementioned analytical results showed that the methodology utilized in our investigation was precise and efficient. In addition, they also proved that antibiotics threw mice's richness and diversity of intestinal flora out of balance, resulting in intestinal-flora instability. The intestinal-flora structure of mice treated with *A. lancea* extract was considerably different from that of animals treated with antibiotics alone. Diversity and abundance of mouse intestinal flora tended to recover, as did the intestinal flora of mice not administered antibiotic injections. Using metabolomics, the impact and implications of these changes will continue to be examined and explored.

### Serum metabolite analysis

**Fig. 10:**
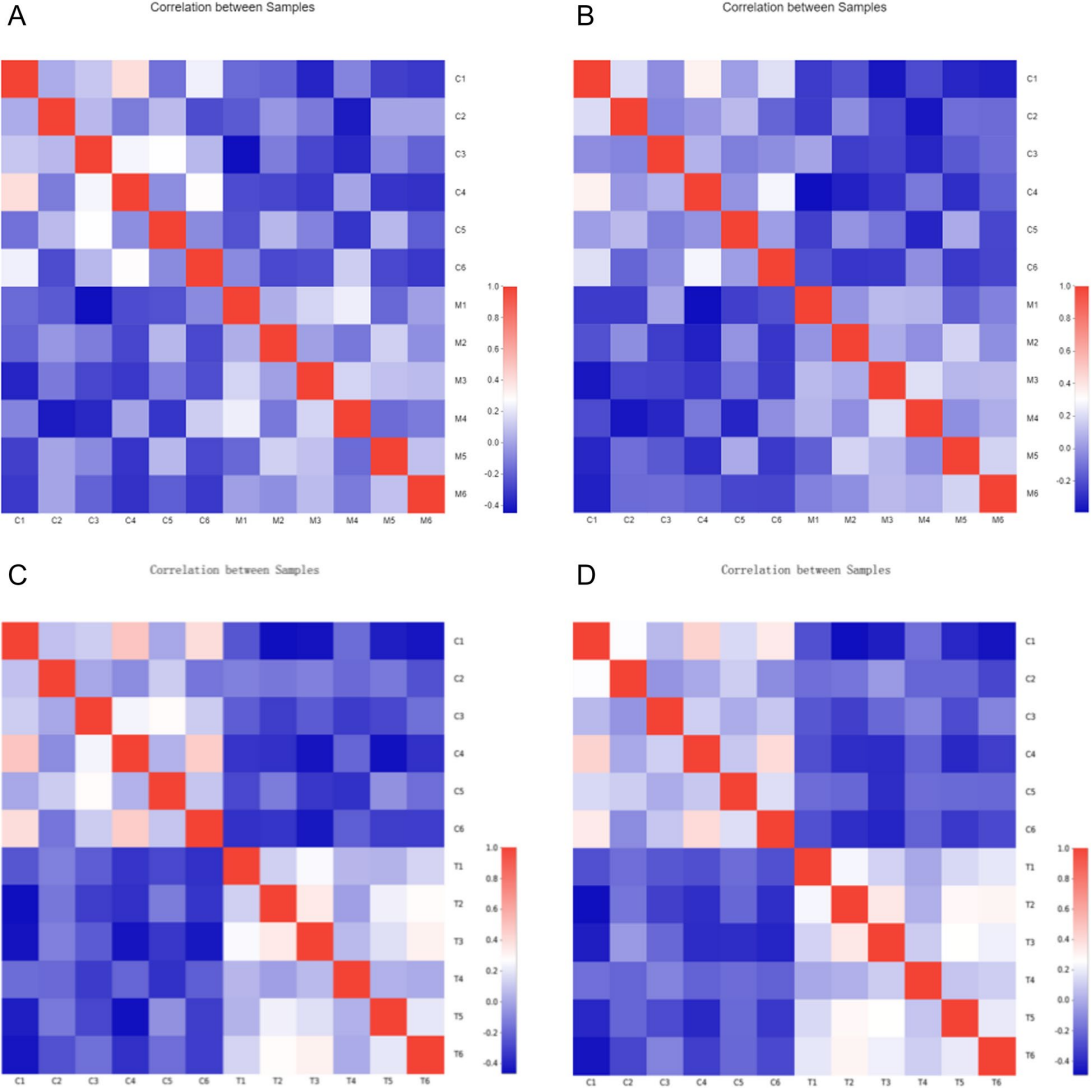
Sample correlation heatmap. **A** Sample correlation heat map in C vs M positive ion mode; **B** Sample correlation heat map in C vs M negative ion mode; **C** Sample correlation heat map in C vs T cationic mode; **D** Sample correlation heat map in C vs T anionic mode

Correlation heatmaps were obtained using Pearson’s correlation coefficient (PCC) and Euclidean distance algorithms in both anionic and cationic modes (Fig. 10). As seen in Fig. 10 and Table 2, there were differences between Groups C and M, particularly in anionic mode. There were also changes between Groups C and T, but these were very minor in cationic mode, and differences were significant in anionic mode.

**Table 2:** Sample correlation coefficient table

	−		+	
Grouping	Average	SD	Average	SD
C *vs.* M	− 0.21211	0.119361	− 0.19895	0.148741
C *vs.* T	0.747097	0.042251	0.820833	0.044537

**Fig. 11:**
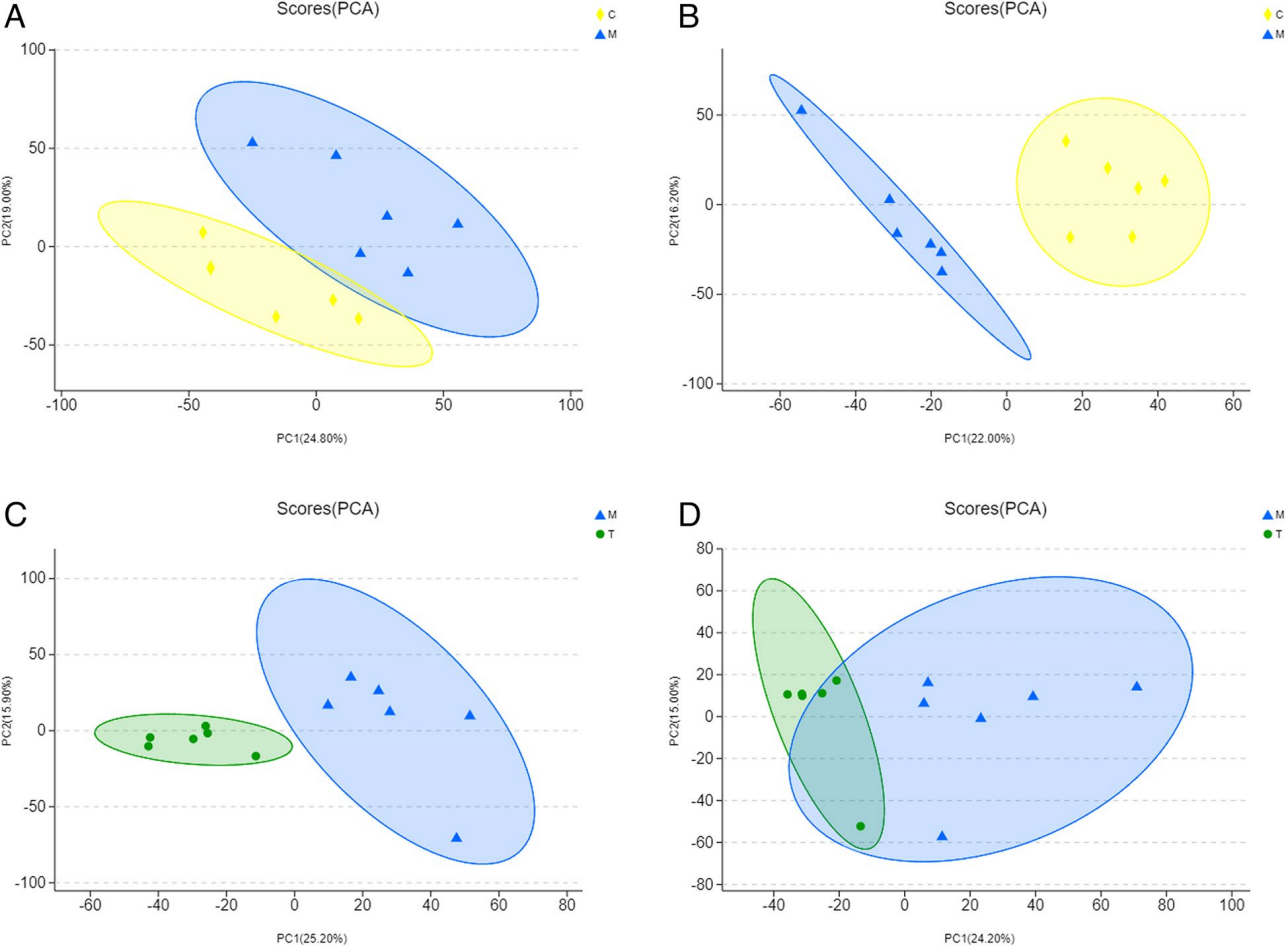
PCA score chart. **A** PCA score of comparison group M vs C in anionic mode; **B** PCA score of M vs C comparison group in cation mode; **C** PCA score of comparison group T vs M in anionic mode; **D** PCA score of T vs M comparison group in cation mode

As shown in the PCA score chart (Fig. 11), we noticed a considerable separation between Group M and other groups in both cationic and anionic modes. In Fig. 11D, the PCA score chart of the T *versus* (*vs.*) M comparison group in cationic mode revealed a partial overlap between Groups T and M, probably because of two-dimensional nature of the exhibited images. Group M was separated from remaining groups.

**Fig. 12:**
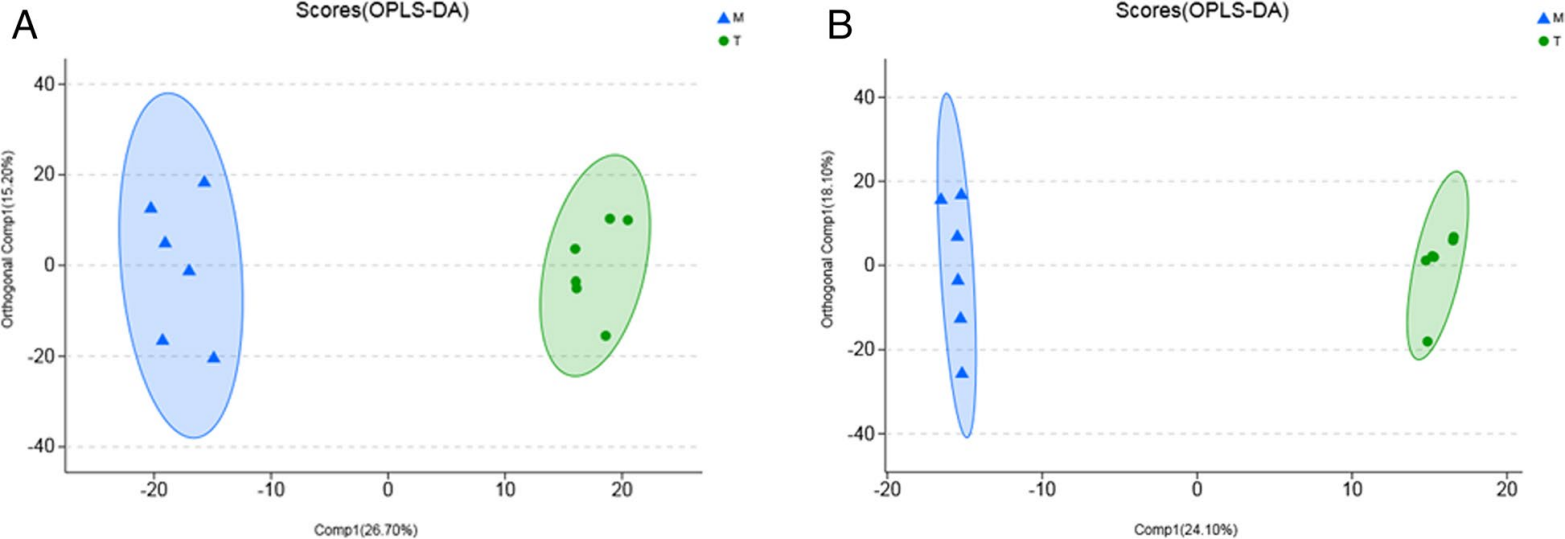
OPLS-DA score chart. **A** opls-da score of M vs T comparison group in anionic mode; **B** pls-da score of T vs M comparison group in cation mode

In addition, additional OPLS-DA model analysis (Fig. 12) also indicated that Groups M and T were distinct, showing that *A. lancea* extract significantly changed the physiological-metabolic condition of mice following antibiotic therapy.

**Fig. 13:**
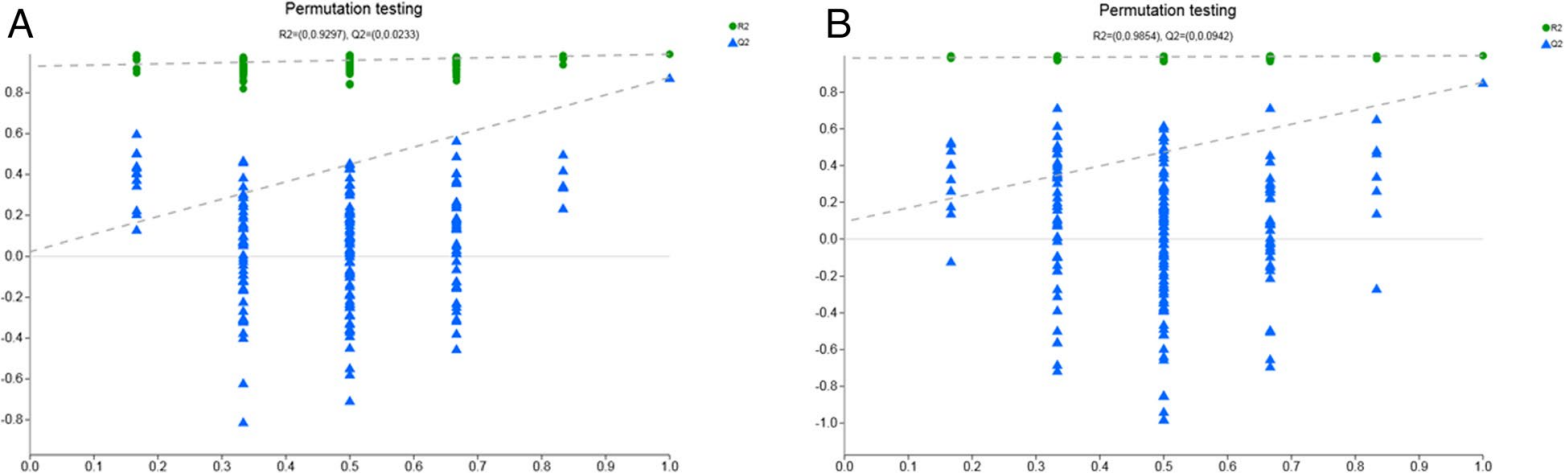
Response permutation test chart. **A** Response permutation test in anion mode; **B** Response permutation test in cation mode

To verify the validity of the OPLS-DA model, we employed response permutation testing to assess its precision (Fig. 13). The main parameters were as follows: *Anionic mode:* R2X (cum) = 0.612, R2Y (cum) = 0.989, Q2 (cum) = 0.827; *Cationic mode:* R2X (cum) = 0.538, R2Y (cum) = 0.994, Q2 (cum) = 0.808. The above parameters are all more than 0.5, R2Y had a high value. This investigation produced a model with great precision, stability, and dependability.

**Fig. 14:**
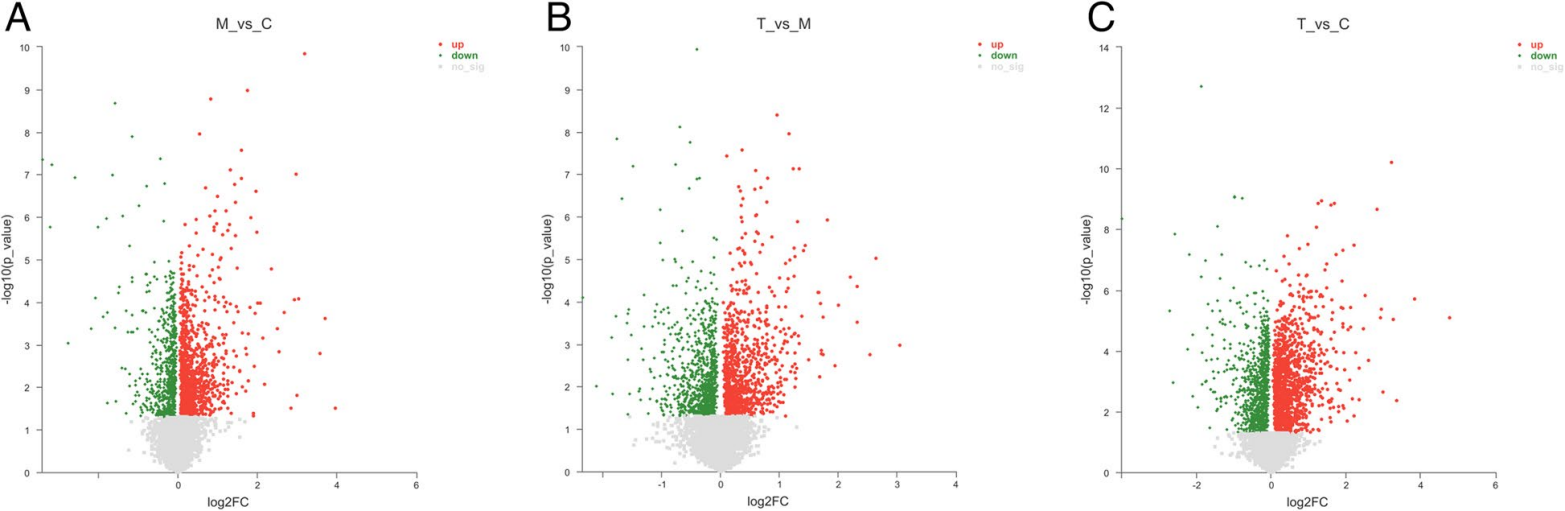
Difference volcano map. **A** M vs C differential volcanic map; **B** T vs M differential volcanic map; **C** T vs C differential volcanic map

Using a volcano map (Fig. 14), we were able to quickly determine the statistical significance of variations in metabolic-expression levels between antibiotics and *A. lancea* extract after gavage and the statistical significance thereof. Figure 14A/14C depicts a multitude of substantially up-regulated regions. Groups T and C differ significantly in up- and down-regulated regions (red and green, respectively) as depicted in Fig. 14C. However, because there is only one value in the red area beyond abscissa 4, this result may have been caused by previously indicated. In addition, the kind of bacteria after Atractylodes lavage was superior to that after antibiotic lavage, and the number of beneficial bacteria was inconsistent with that before lavage. The majority of strongly upregulated and downregulated regions fall within the range of abscissa 1 to 2, with significantly upregulated regions comprising a higher proportion.

The top 30 significantly differential metabolites in the comparison groups (C *vs.* M and M *vs.* T; Tables 3 and 4) was identified using a screening condition of *P* < 0.05, VIP_pred_oPLS-DA > 1, fold change [FC] > 1, or FC < 1. Compared to Group C, the contents of phospholipids such as cephradine, theophylline, lysophosphatidylcholine (LysoPC), and lysophosphatidylethanolamine in Group M were significantly decreased, while the concentrations of 2-phenylglycolic acid, cinnamyl glycine, catechol sulfate, gibanoic acid M, 3-indolepropionic acid, and 5-oxy-ferulic melanin in Group M were significantly increased. Compared to Group M, lipids such as phosphatidylcholine and terpinyl anthranilate were increased in Group T, but emodin, hydroxyl hexadecarboxylic acid, 3-carboxylic 2,3,4,9-tetrahydro1h-pyridine [3,4-B] indole-1-propionic acid, daidzein, and 2-hydroxyundecanoic acid were significantly decreased, indicating that metabolites were significantly changed after antibiotic treatment. *A. lancea* extract in moderation and the number of beneficial bacteria gradually recovered after i.g. administration.

**Table 3:** Top 30 metabolites in Group M *vs* Group C: summary of differential metabolites in the control group

Name	m/z	VIP_pred	FC (M/C)	*P*
Hippuric acid	180.0642	3.2855	1.4692	0.00000001
3-Indolepropionic acid	190.085	4.8292	3.0467	0.00000003
Included acid M	463.3049	3.4779	2.6813	0.00000017
Ethyl maltol	105.0325	3.2351	1.6146	0.00000020
Deoxycholic acid	437.2911	3.4058	1.9847	0.00000032
Cefradine	348.1023	4.1577	0.5041	0.00000054
5-Oxygen-ferulic melanin	452.1549	4.0003	3.5866	0.00000104
Catechol sulfate	188.9862	4.0163	2.4593	0.00000148
Tetradecane diacid	281.1709	1.7161	1.1415	0.00000150
5C-glycoside ligand	273.1145	3.5433	1.8701	0.00000207
Cinnamyl glycine	206.0797	3.7539	2.1163	0.00000909
2-Phenylethanol glucosidic acid	297.098	3.7474	2.0734	0.00000991
Tetracycline	637.3469	1.7577	1.1475	0.00005165
Ganoderic acid H	607.2625	1.8047	1.3637	0.00007320
( ±)12(13)-Epoxy oleic acid	295.2277	1.6249	1.0891	0.00008150
Lysophosphatidylethanolamine (0:0/22:0)	582.3798	1.599	0.9258	0.00009721
Lysophosphatidylethanolamine (0:0/22:1 [13Z])	580.3636	1.5418	0.9221	0.00012840
(E)-10-OXO-8-caproleic acid	183.1024	1.6872	1.2462	0.00014960
(1)-(E)-13-hydroxy-10-OXO-11-octadecenoic acid	277.2152	1.7374	1.1127	0.00015680
Lysophosphatidylethanolamine (0:0/18:2 [9Z,12Z])	522.285	1.6841	0.8144	0.00017630
Phosphatidylcholine (19:1 [9Z]/0:0)	536.3709	1.5516	0.9395	0.00019640
Phosphatidylcholine (17:2 [9Z, 12Z]/0:0)	506.324	1.1043	0.9605	0.00020110
13S-ambrettolic acid	341.2337	2.185	1.3138	0.00021100
Theophylline	649.2492	2.1059	0.7866	0.00032180
Squalane	275.2009	2.2094	1.6423	0.00032480
( ±)12,13-Dihydroxy octadecenoic acid	313.2384	1.5145	1.0802	0.00034500
Lysophosphatidylcholine (20:1 [11Z])	594.3798	1.1581	0.9562	0.00039740
Citric acid	191.0198	1.4411	1.091	0.00047770
(S)-5-Hydroxyl-eicosatetraenoic acid-(Hete)	321.2435	1.5976	1.1383	0.00052270
Pc (22:6 [4z, 7z, 10z, 13z, 16z, 19z]/16:1 [9z])	848.5504	1.4852	0.927	0.00053100

**Table 4:** Top 30 metabolites in Group T *vs* Group M: summary of differential metabolites in the control group

Name	m/z	VIP_pred	FC (T/M)	*P*
RMB 5 c glycosides	273.115	2.7522	0.6965	0.000000018
Rheum emodin	269.045	3.2115	0.3121	0.000000369
DL-2-aminocaprylic acid	160.132	2.4555	1.3267	0.000012090
Malarate	276.199	2.5836	1.3973	0.000046840
(R)-8-acetoxy coumarin acetone	211.132	2.0582	1.2038	0.000053490
5,9-Table dioxane-3-hydroxyergosteride-7-alkene-6-ketone	443.317	2.7401	1.8409	0.000057530
Morindone Q	643.231	3.0719	3.1994	0.000061970
2-E thiophene	199.097	1.8265	1.3509	0.000063960
Hydroxy hexadecarbonate	301.201	2.7644	0.4055	0.000070190
Ficoceryl alcohol	242.247	1.4816	0.909	0.000099410
Phosphatidylcholine (8Z, 11Z, 14Z, 17Z)	810.600	1.2751	1.0362	0.000104800
2,7(14)-Isoprene-10,15-diol	279.160	1.5489	1.1942	0.000150700
Decyl alcohol	200.200	1.432	0.9271	0.000166400
Daidzein	255.064	3.4924	0.4635	0.000230700
Terpinyl o-aminobenzoate	274.182	2.3973	1.2634	0.000295500
( ±)12,13-Dyhydroxy-9z-octadecenoic acid	313.238	1.5429	0.9115	0.000309800
Pteroside Z	415.172	1.8806	0.6016	0.000310500
Dodecyl alcohol	228.231	1.3948	0.9332	0.000316300
( ±)13-Octadecadienoic acid	295.226	1.7853	0.8774	0.000355500
Carboxyl-2,3,4,9-tetralin-1H-pyridine[3,4-b]benzpyrole-1-propionic acid	287.103	2.462	0.4059	0.000384700
Phosphatidylcholine (4Z, 7Z, 10Z, 13Z, 16Z, 19Z)	854.567	1.0789	1.035	0.000412900
Dihydrogen DNA-8-isoamyl alcohol	255.121	2.3496	1.5766	0.000417700
Phosphatidylcholine (7Z, 10Z, 13Z, 16Z)	854.597	1.2123	1.0562	0.000422300
1,17-Heptandiol	314.305	1.4145	0.9137	0.000477900
5(s)-Hete hydroxyl-eicosatetraenoic acid (HETE)	321.243	1.3721	0.8896	0.000499600
Glycoprotein	285.074	2.5292	0.5913	0.000579500
Benzoquinone acetic acid	165.019	2.3902	1.7328	0.000583800
(R)-3-hydroxybutyrate carnitine	248.148	2.2202	1.2947	0.000592300
2-Hydroxyundecanoic acid	201.149	1.9738	0.5553	0.000595400
Baicalin	447.091	2.5655	0.6928	0.000660800

**Fig. 15:**
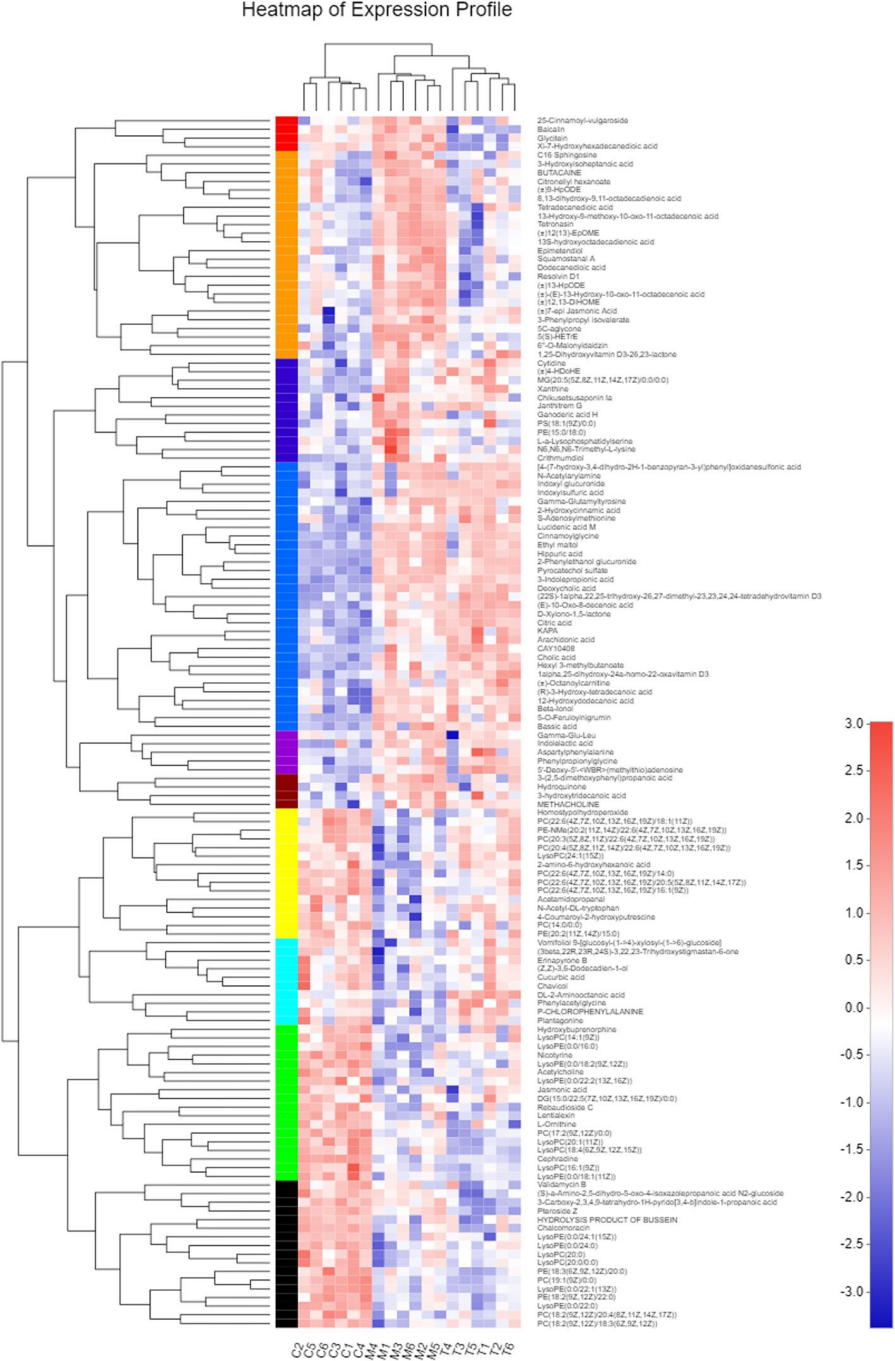
Group M vs Group C cluster analysis of the first 30 metabolites in the control group

**Fig. 16:**
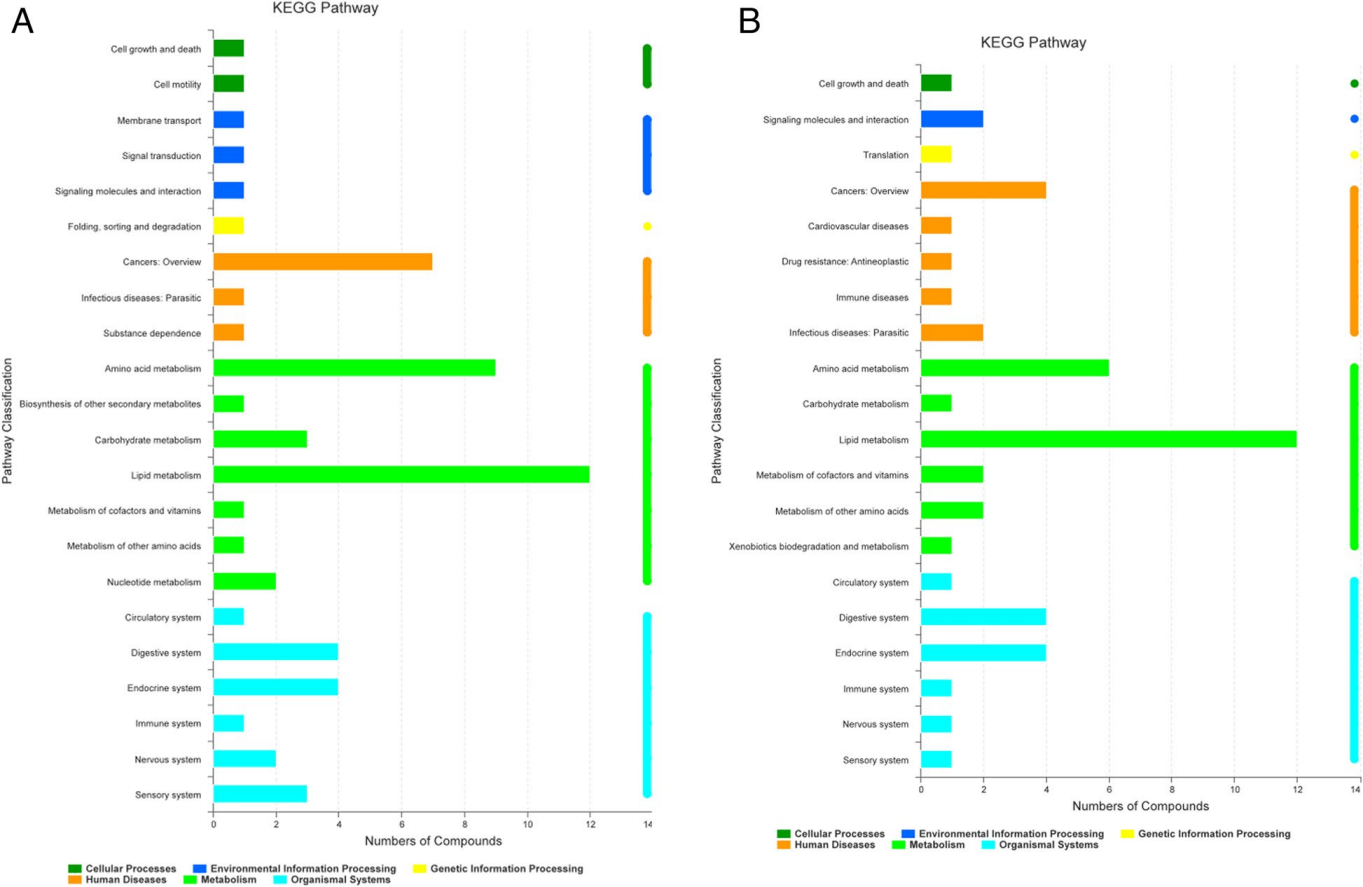
KEGG pathway charts. **A** Group M vs Group C; **B** Group T vs Group M

The cluster analysis of the first thirty metabolites is depicted in Fig. 15. As shown, the expression levels of LysoPC, lysophosphatidylethanolamine, arachidonic acid (AA), and other substances were significantly altered after treatment. The number of metabolites annotated to the lipid metabolism pathway was the greatest, followed by those annotated to the amino acid metabolism pathway (Fig. 16). Differentially annotated lipid metabolites comprise AA, taurocholic acid, traumatic acid, palmityl l-carnitine, dodecanedioic acid, 21-deoxycortisol, 3-oxygen-sulfolactose ceramide, 17-hydroxyprogesterone, 13S-hydroxyoctadecenoic acid, phosphatidylcholine (24:1 [15Z]), phosphatidylcholine (18:3 [6Z, 9Z, 12Z]), and phosphatidylcholine (16:0). The amino acid metabolic pathway were such differential metabolites as phenylacetylglycine, L-methionine, indoleacetaldehyde, thyroxine, gin, and m-coumaric acid.

**Fig. 17:**
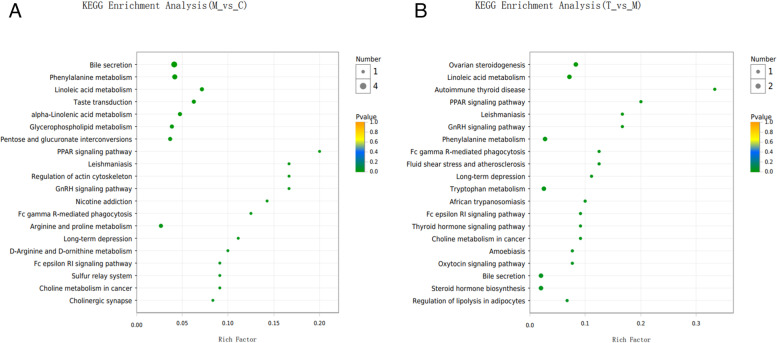
KEGG enrichment analysis. **A** Group M vs Group C; **B** Group T vs Group M

Figure 17 depicts the KEGG enrichment analysis of differential metabolites as a diagram of bubbles. As shown in the enrichment analysis bubble diagram of Group M *vs* Group C, the bubbles in the bile secretion pathway are the greatest, as was the number of metabolites enriched to metabolic concentration (4). The second pathway was phenylalanine metabolism, which was enriched with three metabolites. Two metabolites were enriched for the linoleic acid metabolism, the α-linolenic acid metabolism, the glycerol phospholipid metabolism, the mutual conversion of pentose and glucuronic acid, the arginine metabolism, and the proline metabolism. As seen in the enrichment analysis bubble diagram for Group T *vs.* Group M, the bubbles in linoleic acid metabolism, phenylalanine metabolism, tryptophan metabolism, steroid biosynthesis, bile secretion, ovarian-steroid production, and other pathways are the largest and identical in size, indicating that the number of metabolites enriched to metabolic concentration was equivalent. The ovarian-steroid route has the highest degree of enrichment, followed by linoleic acid metabolism.

**Fig. 18:**
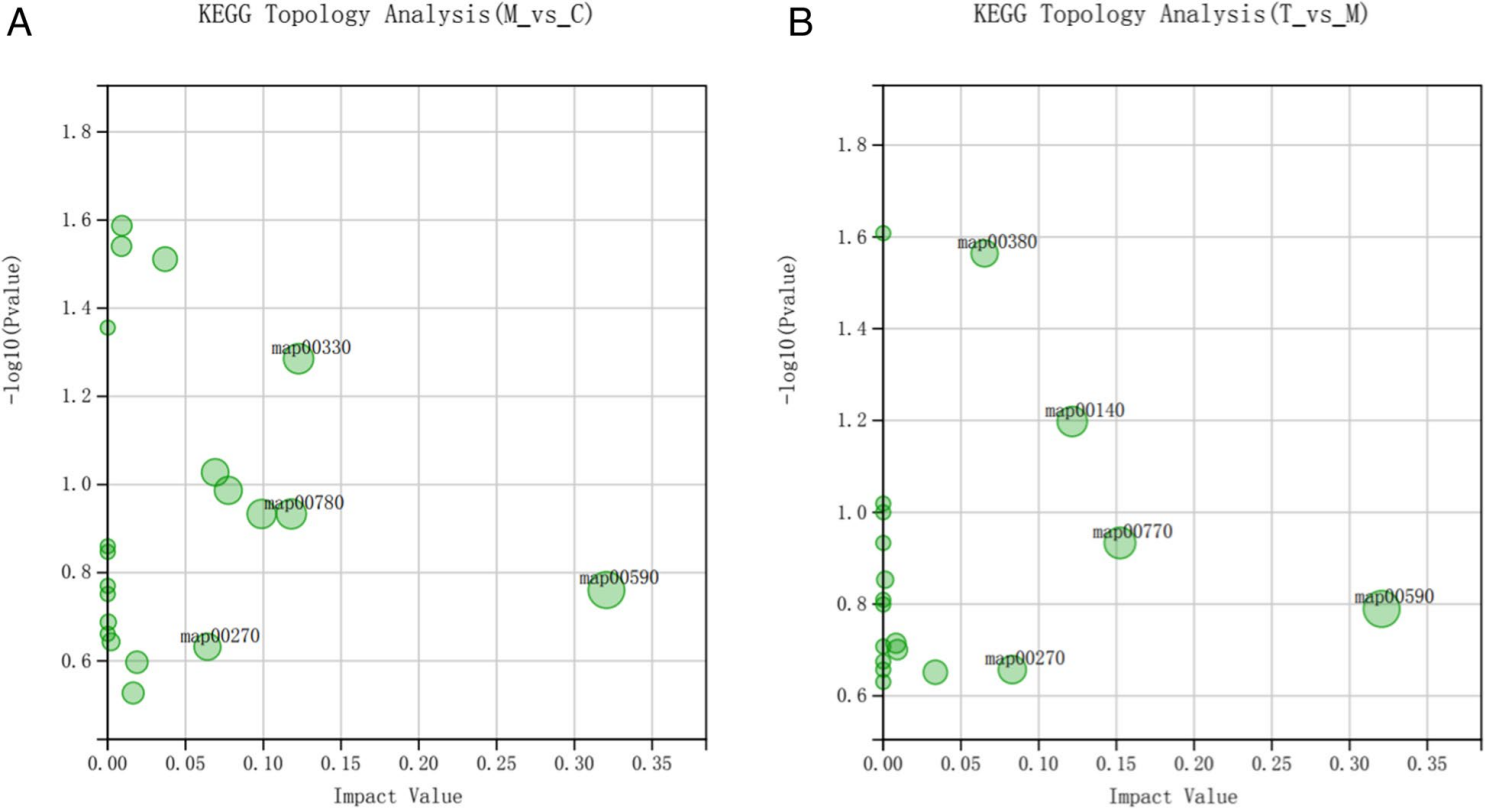
KEGG topology analysis. **A** KEGG Topology Analysis of M vs C; **B** KEGG Topology Analysis of T vs M

**Fig. 19:**
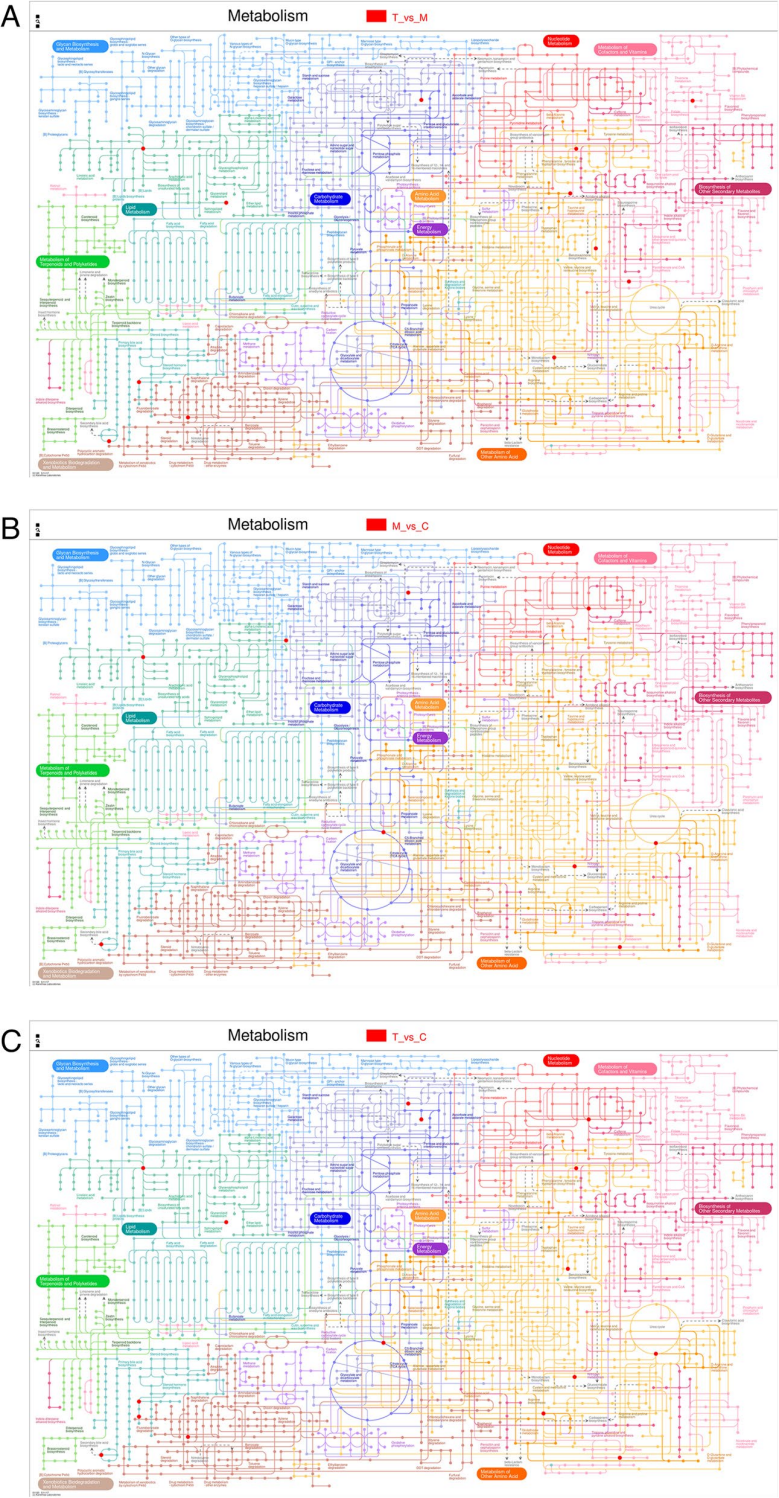
iPath metabolic pathway. **A** iPath metabolic pathway T vs M; **B** iPath metabolic pathway M vs **C**; iPath metabolic pathway of T vs C

According to the KEGG topology analysis bubble diagram of differential metabolites (Fig. 18), the most important KEGG pathway was MAP00590, namely, the AA metabolism pathway. We used Interactive Pathways Explorer (iPath) v3.0 for visual examination of the metabolic pathways of all differential metabolites (Fig. 19). As shown in Fig. 18, lipid metabolism and amino acid metabolism accounted for the majority.

### Correlation analysis between intestinal flora and serum metabolomics

**Fig. 20:**
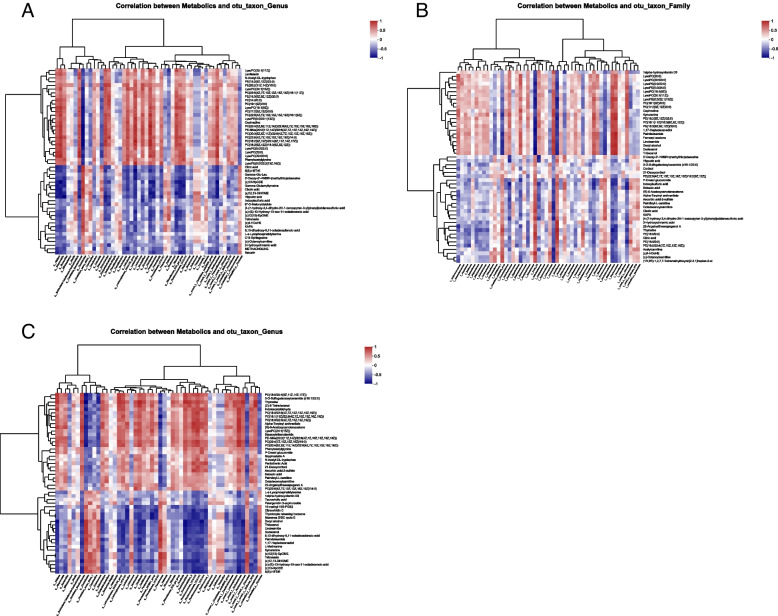
Spearman’s correlation coefficient. **A** M is associated with the C analysis, **B** T is associated with the C analysis, and **C** T is associated with the M analysis

To determine the potential relationship between changes in intestinal flora in feces and changes in metabolites in serum from mice, we compared three conditions and analyzed correlations between differential metabolites in serum and intestinal microflora using Spearman's correlation coefficient (SCC). Figure 20A shows that *G_Odoribacter*, *G_Gordonibacter*, and *Helicobacter* were positively correlated with phenylacetylglycine, lentialexin, and LysoPC. *Staphylococcaceae* and *Streptococcaceae* were positively correlated with phenyluronic acid, lentialexin, and LysoPC, as depicted in Fig. 20B. *Helicobacteraceae* showed a positive correlation with the A group. In the C group, *Odoribacter* was positively correlated with phenylacetylglycine, lentialexin, and LysoPC, just as it was in the A group. *G_Ruminococcaceae* was also positively correlated with these three metabolites, as well as with other species of *G_Ruminococcaceae*. As depicted in Fig. 20, the connection between bacterial species and metabolites in the three groups was broadly comparable, however species differences led to variances in the final results. As a result, we hypothesized that this link was highly dependable and that the metabolites in question could be those of these species or derivatives thereof.

## Conclusions

*A. lancea* is a commonly used TCM in treating GI diseases for its great effect of “clearing dampness”. With the deepening of modern pharmacological researches, increasing researches have demonstrated that *A. lancea* shows a certain effect in alleviating intestinal diseases. For example, it could reduce DSS-induced colitis by regulating intestinal flora and metabolites [29], as well as relieving constipation by regulating intestinal flora [30]. It is clear that intestinal flora plays a crucial role in resulting in GI diseases. What’s more, the components in serum are supposed to affect the types and numbers of intestinal flora [31]. And it is feasible to affect the types and numbers of intestinal flora by using natural products to alleviate or treat diseases [32]. Therefore, in this study, we focus on the intestinal flora and metabolites to study the therapeutic effect of *A. lancea* on GI diseases and explore its underlying mechanisms.

In this study, based on gene sequences of microbiota in the feces of three experimental mouse groups that received different treatments intragastrically, we used 16 s rRNA gene sequencing to detect changes in intestinal microbes and to study whether the extract of the Chinese herbal medicine *A. lancea* had effects on the intestinal flora. The results indicated that when mice were treated with antibiotics (gentamicin and cefradine), their gut flora became less diverse. The dominant species composition of their intestinal flora changed from beneficial to harmful bacteria, and the proportion of Proteobacteria increased. The mice displayed diluted feces, lower activity levels, and rougher hair. By the conclusion of the modeling period, when the mice were administered with *A. lancea* extract, the species composition of their intestinal flora began to restore to normal proportions. Bacteroides and Mycorrhizae colony proportions increased, and microbial equilibrium was restored. The mouse's hair grew silky once more. Normal feces were observed, and their activity level increased.

Based on the results of our metabolomics analysis, we determined that *A. lancea* extract had a significant effect on the lipid and amino acid metabolism of mice. Lipid metabolism is likely reflected in AA metabolism, and amino acid metabolism is most likely reflected in L-methionine metabolism. Lipids, the energy suppliers for organisms, participate in a variety of vital processes, including including the maintenance of cell membrane structure, energy storage, and signal transduction and transport. Linoleic acid is an essential fatty acid that cannot be synthesized by the body and so must be consumed. According to studies, cholesterol in the body can only operate normally when paired with linoleic acid; in the absence of linoleic acid, cholesterol can cause metabolic abnormalities. AA is a ω-6 polyunsaturated fatty acid and a linoleic acid metabolite. Being a chemical with significant biological activity, it plays a critical part in the inflammatory metabolic process. Under conditions of oxidative stress, the conversion of linoleic acid to AA is accelerated. Most inflammatory mediators are created by the metabolism of AA [28]. The cyclooxygenase (COX) and lipo-oxygenase (LO) metabolic pathways can convert AA to prostaglandins (PGs). Inflammation is linked to PGs, prostacyclin (PGI1), thromboxane (TX), and leukotrienes (LT).

Our experimental findings demonstrated that *A. lancea* extract may restore normal levels of possible biomarkers in mice, including hemolysis phosphatidyl choline, L-glutamic acid, AA, deoxycholic acid, and bile acid. The normalization of the index indicates that the intestines of mice eventually recover from metabolic abnormalities. In addition, this extract could be used to treat antibiotic-induced metabolic abnormalities in mice. The mechanism of action may involve the metabolism of AA and phospholipids to increase the level of prostaglandin E2 (PGE2) in the stomach, thereby inhibiting gastric acid output, improving ulcers, and exerting immunosuppressive and anti-inflammatory actions. With the alleviation of the mice's diarrhea, the serum metabolite levels returned to normal. This partially explained the therapeutic impact of *A. lancea* extract and reflected the related changes in the body’s microscopic and macroscopic states during medication therapy. According to the enrichment bubble diagram, the elevated serum fatty acid and amino acid levels of mice in the treatment group suggest that these two metabolic pathways were more active, as was the bile secretion route.

In this study, we effectively developed a mouse model of intestinal-microflora problem induced by antibiotics, and then used 16S rRNA gene sequencing and metabolomics to analyze the regulation mechanism of A. lancea extract on this condition. In these trials, we discovered that A. lancea extracts may partially restore the gut microbiota, the primary and secondary biliary lipid metabolism, and the amino acid metabolism pathways, as well as reverse metabolic abnormalities caused by antibiotic therapy. We believe that this study will provide new medicinal concepts for advancing our understanding of the pathophysiology of intestinal inflammation and the early detection of intestinal microbiota imbalance.
